# Protein genes in repetitive sequence—antifreeze glycoproteins in Atlantic cod genome

**DOI:** 10.1186/1471-2164-13-293

**Published:** 2012-07-02

**Authors:** Xuan Zhuang, Chun Yang, Svein-Erik Fevolden, C-H Christina Cheng

**Affiliations:** 1 Department of Animal Biology, University of Illinois, Urbana-Champaign, Illinois, 61801, USA; 2 Department of Molecular and Integrative Physiology, University of Illinois, Urbana-Champaign, Illinois, 61801, USA; 3 Currently at EMD Millipore, San Diego, CA, USA; 4 Department of Arctic and Marine Biology, University of Tromsø, N-9037, Tromsø, Norway

## Abstract

**Background:**

Highly repetitive sequences are the bane of genome sequence assembly, and the short read lengths produced by current next generation sequencing technologies further exacerbates this obstacle. An adopted practice is to exclude repetitive sequences in genome data assembly, as the majority of repeats lack protein-coding genes. However, this could result in the exclusion of important genotypes in newly sequenced non-model species. The absence of the antifreeze glycoproteins (AFGP) gene family in the recently sequenced Atlantic cod genome serves as an example.

**Results:**

The Atlantic cod (*Gadus morhua*) genome was assembled entirely from Roche 454 short reads, demonstrating the feasibility of this approach. However, a well-known major adaptive trait, the AFGP, essential for survival in frigid Arctic marine habitats was absent in the annotated genome. To assess whether this resulted from population difference, we performed Southern blot analysis of genomic DNA from multiple individuals from the North East Arctic cod population that the sequenced cod belonged, and verified that the AFGP genotype is indeed present. We searched the raw assemblies of the Atlantic cod using our *G. morhua* AFGP gene, and located partial AFGP coding sequences in two sequence scaffolds. We found these two scaffolds constitute a partial genomic *AFGP* locus through comparative sequence analyses with our newly assembled genomic *AFGP* locus of the related polar cod, *Boreogadus saida.* By examining the sequence assembly and annotation methodologies used for the Atlantic cod genome, we deduced the primary cause of the absence of the AFGP gene family from the annotated genome was the removal of all repetitive Roche 454 short reads before sequence assembly, which would exclude most of the highly repetitive AFGP coding sequences. Secondarily, the model teleost genomes used in projection annotation of the Atlantic cod genome have no antifreeze trait, perpetuating the unawareness that the AFGP gene family is missing.

**Conclusions:**

We recovered some of the missing AFGP coding sequences and reconstructed a partial *AFGP* locus in the Atlantic cod genome, bringing to light that not all repetitive sequences lack protein coding information. Also, reliance on genomes of model organisms as reference for annotating protein-coding gene content of a newly sequenced non-model species could lead to omission of novel genetic traits.

## Background

Massively parallel deep coverage next generation sequencing (NGS) technologies have stimulated efforts of *de novo* genome sequence assembly in recent years. While NGS data productions advance at phenomenal rates, accurate genome assembly and annotation remain challenging, and the extent of what may be missing in these *de novo* assembled genomes is an ongoing matter of concern. All genome assembly efforts face the challenge of accurately assembling tandem and sparse repeat sequences. Current assembly algorithms collapse identical or very similar repeats leading to potential reduction or loss of genomic complexity [1]. The short reads produced by current NGS technologies are especially prone to this problem, as repetitive sequences can be resolved only if the reads are long enough to span the repetitive region [2]. A frequent practice is to exclude highly repetitive sequences in genome assembly and annotation, on the assumption that they lack protein-coding genes. While this assumption is generally valid, here we provide a clear example that excluding what appears to be simple repetitive sequences could result in the exclusion of an important fitness genotype, in the case of the Atlantic cod genome.

Atlantic cod is a key commercial fishery species in the cold waters of the north Atlantic seas and a prime target for domestication by countries across the north Atlantic Ocean. Star et al. [3] recently reported the annotated genome of a specimen (NEAC_001) from the cold-adapted North East Arctic cod (NEAC) stock from the Barents Sea. The NEAC_001 genome was among the first complex vertebrate genomes assembled entirely from short reads obtained using the Roche 454 GS FLX Titanium platform. The report discussed the interesting potential thermal adaptive properties of the hemoglobins of the cod, as well as its unique adaptive immune system as related to organismal fitness [3]. While not the focus of the discussion, it is nevertheless surprising that a crucial fitness trait, the antifreeze glycoprotein (AFGP) [4-6], which has clear relevance for aquaculture of the species in cold, northerly latitudes, was absent from genome annotations and predicted transcripts and proteins. Neither was any allusion made to its presence in the extensive Supplementary Notes accompanying the report.

AFGP is one of the diverse, novel antifreeze proteins that evolved in various polar and subpolar marine teleost lineages, enabling their survival in freezing, icy seawater [7,8]_._ Presence of AFGPs is long known in a number of northern and Arctic Atlantic cod populations [4-6]. The near-identical AFGPs in the Arctic/northern cods (family Gadidae) and in the unrelated Antarctic notothenioid fishes endemic to the Southern Ocean, is an established prime example of convergent evolution and at the rare protein sequence level [9]. Antifreeze proteins recognize environmental ice crystals that enter the fish, bind to them and stop their expansion, thereby prevent the fish body fluids, which have less salt and thus a higher freezing point than seawater, from freezing [10]. Absence of the AFGP genotype in the cold-adapted NEAC_001 would seem inconsistent with its frigid Arctic habitats.

AFGPs are highly repetitive in sequence in the protein and particularly in the coding sequences because they are encoded as large polyprotein precursors [9]. Thus the possibility exists that the repetitive AFGP coding sequence repeats might have been inadvertently excluded along with other non-protein coding repetitive sequences during genome sequence assembly. Here we report investigations of the Atlantic cod genome data leading to our discovery that AFGP coding sequences exist in NEAC_001. We provide experimental evidence as well as bioinformatics proofs from comparative genomic sequence analyses, which support the presence of an AFGP gene family in Atlantic cod. From examining in detail the published methodologies in the Atlantic cod genome assembly and annotation process, we deduced the probable causes leading to the exclusion of this major genetic trait.

## Results and discussion

### AFGP gene family in Atlantic cod

**Figure 1:**
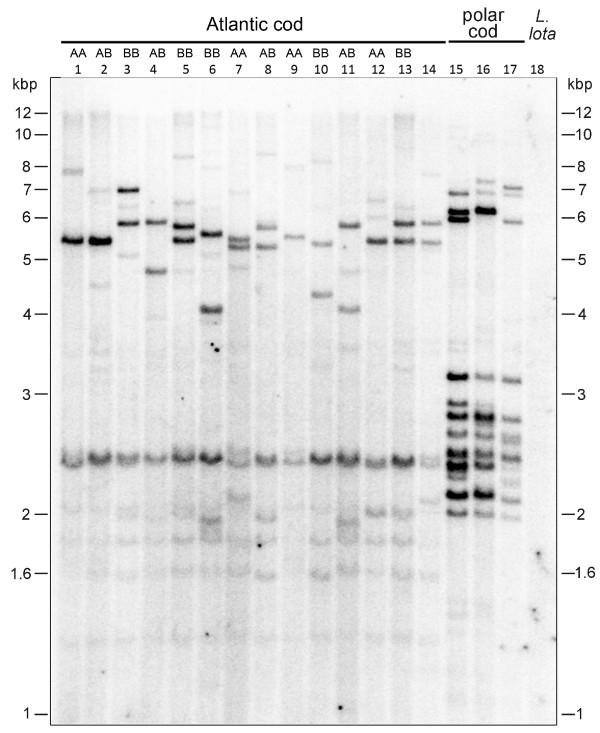
**Southern blot analysis of Atlantic cod genomic DNA showing presence of AFGP coding sequences.***Taq*I digested genomic DNA (~10–15 μg) from Atlantic cod (lanes 1–14) and polar cod (lanes 15–17) hybridized strongly to a polar cod *B. saida* AFGP coding sequence probe. Atlantic cod individuals include Norwegian coastal cod (NCC) and North East Arctic cod (NEAC) from the Finnmark coast and marginal Barents Sea sites: (lanes 1–3) N69° 26.91′ E19° 37.56′; (lanes 4–5) N69° 58.34′ E30° 2.37′; (lanes 6–8) N70° 7.24′ E30° 48.47′; (lanes 9–13) N71° 11.93′ E27° 59.29′, and one individual (lane 14) from Øresund, Denmark. NEAC and NCC are distinguished by their *Pan*I genotype, BB and AA respectively as indicated, while AB can either be NEAC or NCC. For comparison, the related freshwater cod *Lota lota* (lane 18) that does not have AFGP shows no hybridization.

To experimentally verify the presence of the AFGP genotype specifically in the North East Arctic cod (NEAC) and Norwegian coastal cod (NCC), used for genome sequencing and BAC library support respectively [3], we performed Southern blot analysis of genomic DNA of multiple individuals from these two populations (Figure 1). These individuals were collected from several sites along the Finnmark coast (the extreme northeast coast of Norway) and marginal Barents Sea. We identified them as NEAC or NCC based on the specimen’s pantophysin I (*Pan*I) genotype, the accepted marker for distinguishing the two stocks [11-13]. The NEAC stock is homozygous (BB) for the presence of an internal *Dra*I restriction site in its *Pan*I, while the NCC is homozygous for the absence (AA), and heterozygous individuals (AB) can be from either stock but more likely NCC [12]. All NEAC and NCC individuals we examined showed multiple strong AFGP-hybridizing bands to an AFGP coding sequence probe derived from the related, fully AFGP-fortified polar cod *Boreogadus saida*[9], similar to the hybridization in the three polar cod individuals included as positive controls (Figure 1). The repetitive coding sequence (cds) of the AFGP polyprotein precursor is encompassed in a single exon for known gadid AFGP genes [9]; this study], and does not contain recognition sequence sites for the restriction enzyme *Taq*I used for the genomic DNA digests. Therefore, each hybridizing band (*Taq*I fragment) in the genomic Southern blot represents one or more AFGP genes, indicating that a family of AFGP genes or highly similar sequences are present in the genome of both NEAC and NCC. Furthermore, we constructed a partial genomic DNA library for a *G. morhua* individual from outer Øresund, Denmark, and isolated and characterized an AFGP gene named Gm1-1 [Genbank:AF529262]. We also quantified the circulating AFGPs to be about 5 mg/mL in the blood serum of individuals from the Øresund population (results not shown), supporting the presence of functional AFGP genes in Atlantic cod.

### *AFGP* locus in Atlantic cod genome

We envisioned that AFGP cds would still be in the raw genome data of the sequenced NEAC_001, thus we BLAST searched the raw assembly ATLCOD1A with Gm1-1 *AFGP* sequence as query. (ATLCOD1A was assembled with Newbler, and subsequently repeat-masked and used for projection genome re-ordering and annotation. The search yielded sequence similarities in two sequence scaffolds, ATLCOD1As00125 and ATLCOD1As03479. We identified a total of seven partial AFGP genes/coding regions in these two scaffolds—five in ATLCOD1As00125 and two in ATLCOD1As03479. The majority of the repetitive AFGP cds was missing, thus these AFGP genes contain gaps in the middle, but the available sequence lengths at the ends flanking the gaps are sufficient for gene identification. An alignment of the ATLCOD1A partial AFGP cds with the full AFGP coding region in Gm1-1 AFGP gene is shown in Additional file 1.

Mature AFGPs occur as a family of size isoforms composed of four to tens of repeats of the tripeptide (Ala/Pro-Ala-Thr), with glycosylation (the disaccharide galactose-N-acetylgalactosamine) on the Thr residues [9]. The alignment (Additional file 1) shows that the partial AFGP genes of NEAC_001 encode the characteristic tandem tripeptide repeats of the AFGP peptide backbone, as well as the conserved C-terminus sequence, AAAVL*. The aligned nucleotide sequences between ATLCOD1A_AFGP5 and Gm1-1 are 99.8% identical, thus the two are quite clearly counterparts of each other in NEAC_001 and the Øresund individual. These high sequence identities indicate that at least some of the NEAC_001 partial AFGP genes we identified from the two ALTCOD1A sequence scaffolds are intact/functional genes.

**Figure 2:**
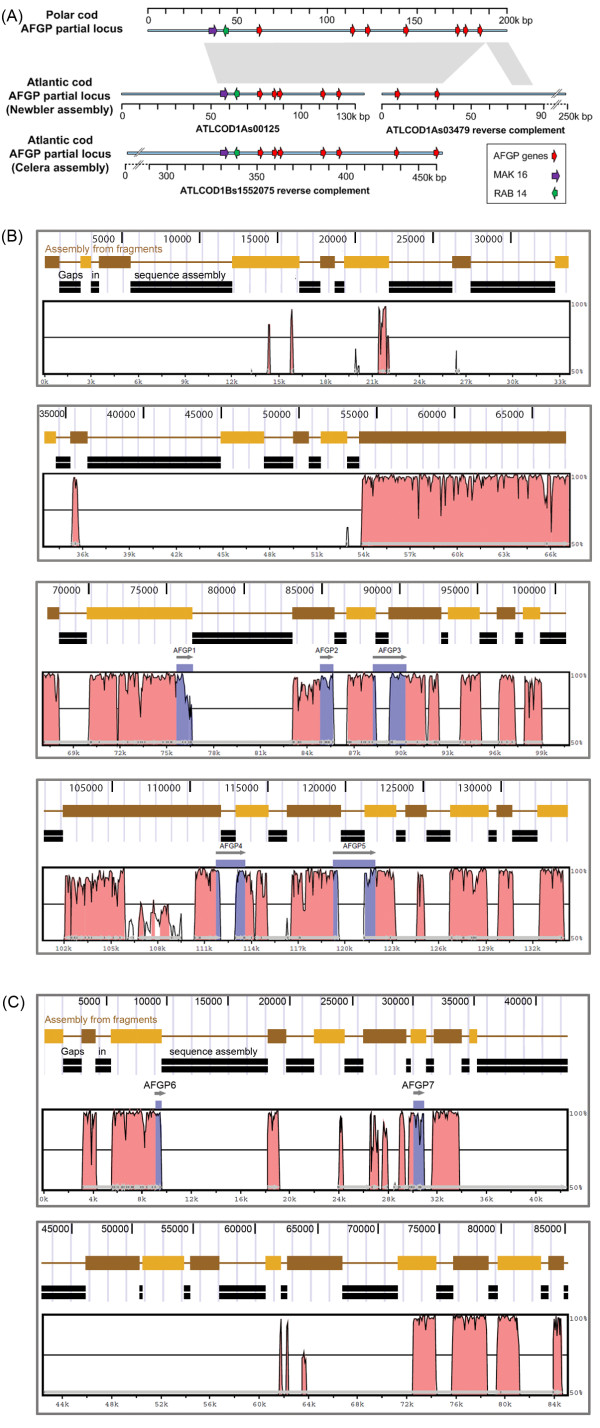
**Alignment of AFGP-containing scaffolds in Atlantic cod and partial AFGP genomic locus from polar cod.** (**A**) Schematic alignment map—Grey shaded areas indicate regions of high nucleotide identities between polar cod and Atlantic cod. AFGP partial locus of Atlantic cod is represented by two sequence scaffolds (ATLCOD1As00125 and ATLCOD1As03479) in the Newbler assembly (ATLCOD1A), and one sequence scaffold (ATLCOD1Bs1552075) in the Celera assembly (ATLCOD1B). AFGP genes of polar cod and fragmented coding sequences in the cod scaffolds are depicted as red arrows pointing towards the 3′ end. Hypothetical protein-coding genes MAK16-like and RAB14-like are denoted as purple and green arrows, respectively. (**B**, **C**) Nucleotide alignments determined using VISTA—The two Atlantic cod sequence scaffolds ATLCOD1As00125 (**B**) and ATLCOD1As03479 (**C**) are depicted as connected brown and orange rectangular bars (assembled sequence fragments) and double black bars (gaps between the sequence fragments), generated with UCSC Genome browser. The framed histogram below the Atlantic cod sequence scaffold structures was VISTA generated plots of their alignment with the polar cod partial AFGP locus sequence. Purple areas in the histogram denote conserved AFGP sequences, and grey arrows denote the AFGP genes we annotated in the two Atlantic cod scaffolds. Pink areas denote other conserved regions. In (**B**) the full length of ATLCOD1As00125 (134,272 bp) was shown. In (**C**), the first 85 kbp of ATLCOD1As03479 (254,310 bp, reverse complement sequence) was shown; the remaining region of the scaffold did not align with polar AFGP genomic region and therefore not shown. The conserved sequence blocks between Atlantic cod and polar shared very high sequence identities ranging from 80% to 99%, indicative of shared microsynteny.

To determine the spatial relationship of the two AFGP-containing ATLCOD1A scaffolds, we compared their sequences to the genomic *AFGP* locus of the related polar cod *B. saida* currently being reconstructed from BAC clone sequences in our laboratory. The two ATLCOD1A sequence scaffolds aligned with a ~200 kbp portion of our polar cod *AFGP* locus assembly [GenBank:JN828577]. The spatial alignment map of the two ATLCOD1A scaffolds with the polar cod *AFGP* partial locus is shown in Figure 2A, and the nucleotide alignments in the form of sequence identity plots using VISTA [14] are shown in Figure 2B, C for the two scaffolds respectively. The extensive conserved regions between the two cods included both AFGP coding sequences and intergenic sequences, and they share high nucleotide identities (~80–99%), indicating they are microsyntenic regions. The microsynteny is further supported by two hypothetic protein coding genes (RAB 14-like and MAK 16-like) shared by the two species in the nearby upstream region to their respective *AFGP* locus (Figure 2A). We also identified a third *AFGP*-containing scaffold, in the ATLCOD1B/Celera assembly (not used for genome annotation), which spans the gap between the two ATLCOD1A scaffolds, confirming they are collinear (Figure 2A).

Together, the results from the genomic Southern blot (Figure 1) and comparative sequence analyses (Additional file 1 and Figure 2) clearly support an *AFGP* genomic locus with intact genes is present in the sequenced NEAC_001.

### Possible cause of *AFGP* exclusion from the annotated cod genome

Through detailed examination of the assembly and annotation process described by Star et al. [3] in their Supplementary Notes, we deduced the possible cause of *AFGP* exclusion in the annotated Atlantic cod genome to be two-fold. The primary cause is the removal of repetitive sequences in the initial steps of the bioinformatics pipeline, and secondarily due to the use of genomes of non-AFGP bearing model teleosts as reference for annotating protein gene content in Atlantic cod.

AFGP sequences are repetitive in the protein and highly repetitive in the encoding gene [9]. Gadid AFGPs are encoded as a large polyprotein precursor containing multiple AFGP molecules that are cleaved post-translationally at Arg or Lys residues that occasionally replace Thr in the Ala/Pro-Ala-Thr tripeptide repeats, to produce the different AFGP size isoforms (Additional file 1, [9]). Thus the AFGP polyprotein coding sequence region, encompassed in a single large exon, consists of a long run of repetitive 9-nucleotide (three codons) sequences that resemble short tandem repeats (STRs). As illustration, the single coding exon of the Atlantic cod AFGP polyprotein in Gm1-1 is ~1. 3 kbp, and contains 141 three-codon (9 nucleotides) repeats encoding 141 tripeptide repeats. A tally of the codon usage (Table 1) shows pronounced bias, resulting in a predominant 9-nt sequence of GC(C/G/A)-GCC-AC(A/T), which will appear to assembler algorithms as a very long string of 9-nt STRs. Also because of the codon bias, this repeated 9-nt sequence can be further reduced into a 3-nt equivalent of the predominant sequence (G/A)C(C/A) (Table 2), which will likely be regarded by assembly programs as trinucleotide simple sequence repeats (SSRs). Escalating the repetitiveness is that gadid AFGP polyprotein genes have duplicated under selection from frigid marine conditions, forming multigene families (Figure 1) that would meet the demand for an abundance of the survival protein. The two ATLCOD1A sequence scaffolds alone contain seven AFGP genes (Figure 2), and potentially more if additional genes exist within the gaps. This expectedly will result in a plethora of short repetitive AFGP coding sequences in the deep-coverage raw Roche 454 reads.

**Table 1:** **Codon usage bias in the 141 9-nt tripeptide repeat coding sequences in *G. morhua* AFGP gene Gm1-1**

	**Codon 1 (Ala/Pro)**			**Codon 2 (Ala)**			**Codon 3 (Thr/Arg)**		
**nt/position**	**1**	**2**	**3**	**4**	**5**	**6**	**7**	**8**	**9**
G	122	0	45	141	0	0	2	4	0
A	0	0	45	0	0	20	139	0	68
C	19	141	49	0	141	121	0	137	25
T	0	0	2	0	0	0	0	0	48
Predominant nt	G	C	C/G/A	G	C	C	A	C	A/T
%	86.5	100	34.6/31.9/31.9	100	100	86.4	98.6	97.2	48.2/34.0

**Table 2:** **Trinucleotide equivalent of the biased 9-nt tripeptide repeat coding sequences in *G. morhua* AFGP gene Gm1-1**

**Single codon/3-nt equivalent (Ala/Pro/Thr/Arg)**			
**nt/position**	**1**	**2**	**3**
G	265	4	45
A	139	0	133
C	19	419	195
T	0	0	50
Predominant nt	G/A	C	C/A
%	62.6/32.9	99.1	46.1/31.4

Note: There are a total of 423 single codons from the 141 9-nt (3-codon) repeats in Gm1-1.

Supplementary Note 5 of Star et al. [3] indicated that before assembling the Roche 454 reads, they “excluded highly repetitive, non-informative reads” from their data set. They stated that these included shotgun reads encompassing STRs and SSRs, and sets of paired-end reads if one or both ends consist of STRs or SSRs. This data reduction likely completely eliminated the STR- or SSR-like AFGP cds except for the 5′ and 3′ ends of the AFGP coding exon immediately adjacent to non-repetitive upstream and downstream sequence (Additional file 1), leading to gaps in the middle of AFGP genes we identified in the two ATLCOD1A scaffolds (Additional file 1; Figure 2B, C). The STR/SSR-culled read data were assembled using Newbler and the Celera assembler, generating ATLCOD1A and ATLCOD1B respectively (Supplementary Notes 5, 6 of [3]). The Newbler assembly ATLCOD1A was chosen for genome annotation. Repeat masking was applied to ATLCOD1A using existing TE (transposable elements) libraries and a *de novo* created custom library for cod, (Supplementary Note 16 and Supplementary Table 6 of [3]) resulting in the masking of 25.4% of the assembly. The repeat-masked ATLCOD1A then underwent whole-genome structural alignment and re-ordering using the three-spined stickleback genome as reference followed by projection annotation Supplementary Note 17 of [3]. We compared the unmasked and repeat-masked version of ATLCOD1A, and found most of the AFGP cds that survived the STR/SSR culling (shown in Figure 2B, C) became masked (highlighted in Additional file 1), rendering the AFGP genotype essentially non-existent prior to protein gene annotation. Annotation was carried out by projecting protein-coding gene models from three-spined stickleback through the whole genome alignments onto the re-ordered cod genomic regions. Additional protein-coding gene models from other teleost genomes (medaka and zebrafish) were mapped onto cod genome regions having no alignment with stickleback (Supplementary Note 17 of [3]). None of these model teleosts require antifreeze protection in their temperate or tropical habitats and had not evolved the novel antifreeze genotype, thus, the exclusion of a major genetic trait in the assembled Atlantic cod genome remained unrecognized.

### Gene-rich repetitive sequences

Exclusion of the well-known prominent and important AFGP trait in Atlantic cod genome annotation brings to light that common assumptions of repetitive sequences as gene-less or gene-poor do not always apply. Atlantic cod AFGP genes are by no means a lone case of gene-rich repetitive sequences representing a major and/or novel trait. Other prominent proteins composed of short repetitive sequences are present in a variety of organisms, including the convergently evolved AFGPs in the Antarctic notothenioids [9,15], other antifreeze proteins in fish [16], insects [17] and plants [18], fibrous silk fibroins in spiders [19] and moths [20], amelogenin in primates [21], human dentin sialophosphoprotein [22], involucrin [23], collagens [24], and others. Exclusion of the repetitive coding sequences of these prominent proteins from the genome assembly of the respective organism would be a major blunder. Missing repetitive and duplicated sequences due to limitations in sequence assemblers has also resulted in omission of more subtle coding exons in human genomes despite the availability of a highly refined reference genome [25,26].

## Conclusions

We have “resurrected” some of the missing AFGP genes from the Atlantic cod raw genome assemblies, and reconstructed a partial genomic *AFGP* locus for this species. While it is well appreciated that all genome assemblies have gaps of information due to bioinformatics limitations, missing a known and prominent fitness trait lends to confusion in the field. We therefore suggest that this biologically relevant trait be restored to the cod genome annotations. The gadid AFGP trait is relevant not only to the biology and culturing potential of the cod species, but as an evolutionary innovation, has broad relevance in the pursuit of understanding molecular mechanisms of invention of new gene and function. For the ever expanding efforts at *de novo* assembly of new genomes, the case of the missing Atlantic cod AFGP genotype hopefully will promote vigilance in avoiding categorical assumptions that all repetitive sequences lack protein coding information. While we await improved algorithms for accurately assembling repetitive sequences, longer reads from traditional sequencing methods such as Sanger still has its place and the painstaking approach of assembling repeat sequences with extensive manual inspection and validation is unavoidable. This approach has been successfully applied to assemble the ~400 kbp highly repetitive and polymorphic AFGP genomic locus that convergently evolved in the Antarctic notothenioid fish [15]. Lastly, while projection annotation of *de novo* assembled genomes of new species using model genomes as reference certainly has great utility, novel traits might have evolved since the species diverged. Thus an appreciation of the evolutionary history and major known biological traits of a new species targeted for genome sequencing is inevitably necessary.

## Methods

### Specimens, DNA isolation and Southern blot hybridization

Atlantic cod *G. morhua* individuals of the Norwegian coastal cod (NCC) stock and North East Arctic cod (NEAC) stock were caught by trawl from the Finnmark coast and marginal Barents Sea sites. Individuals from outer Øresund, Denmark were caught with hook and line. Polar cod *B. saida* were obtained by trawling near Spitzbergen, and the fresh water cod *Lota lota* that does not have the AFGP trait was obtained from Oneida Lake, New York. DNA was isolated from liver or gill tissues using standard Tris.HCl/SDS lysis and phenol/chloroform extractions. About 10–15 μg of *Taq*I (NEB) digested DNA was vacuum blotted onto Hybond-N membrane (GE Health Science). Hybridization to a P^32^-labeled *B. saida* AFGP coding sequence probe (AFGP gene Bs3-1, [9]) was carried out in PerfectHyb (Sigma) at 55°C. The blot was washed thoroughly in 0.1XSSC/0.5%SDS at 55°C and autoradiographed using a phosphor storage screen and the phosphoimager STORM (Molecular Dynamics).

### *Pan*I genotyping of NEAC and NCC

A 773-base pair fragment of the pantophysin gene was PCR-amplified from DNA and scored for the presence or absence of a *Dra*I site, representing the *Pan*I^B^ and *Pan*I^A^ allelic classes respectively, following published protocol [11].

### Isolation and sequencing of Øresund *G. morhua* AFGP gene

A partial genomic DNA library enriched for AFGP genes was constructed using the λZAP Express vector (Stratagene). Genomic DNA was completely digested with *Mbo*I, and DNA fragments within the size range that hybridized to the P^32^-labeled *B. saida AFGP* probe as determined in separate Southern blot experiments were recovered from agarose gel and ligated to the compatible ends of *Bam*HI digested λZap Express vector. The ligation was packaged using Gigapack Gold III (Stratagene) to form the phage library, which was then screened with the *AFGP* probe by plaque lift filter hybridization. Positive phage clones were screened to homogeneity and excised to produce the phagemid (pBK-CMV) DNA with the ExAssist helper phage (Stratagene). One phagemid clone Gm1-1 was selected for sequencing. A nested set of unidirectional deletion clones of Gm1-1 phagemid DNA containing the repetitive AFGP cds was generated using the Erase-a-Base system (Promega), and sequenced using BigDye v2.0 chemistry (ABI).

### Isolation and sequencing of *B. saida* AFGP genomic locus

A BAC (Bacterial Artificial Chromosome) genomic DNA library was constructed for a *B. saida* individual following published protocols [15,27] with modifications. Briefly, agarose-plug immobilized red blood cell DNA was treated with CTAB (cetyl trimethylammonium bromide) (Teknova) to remove blood cell glycoproteins. The treated plugs were then partially digested with *EcoR*I in the presence of *EcoR*I methylase and resolved on pulsed field electrophoresis using CHEF Mapper XL (BioRad). Size fragments of 100–200 kbp were electroeluted and ligated to the *EcoR*I digested pCC1BAC vector (Epicentre), and the ligation was electroporated into *E. coli* DH10B (Invitrogen). Recombinant clones were robotically archived and printed on nylon hybridization membrane as macroarray. The arrayed library was screened with the P^32^-labeled Bs3-1 *AFGP* probe. Details of library statistics and fingerprinted contig analyses of AFGP-positives will be published elsewhere. The partial AFGP genomic locus used in this study comprised of two overlapping AFGP-positive BAC clones that were sequenced using both shotgun libraries constructed with the Nextera kit (Epicentre) and 3-kbp paired-end libraries, on the Roche 454 GS FLX Titanium platform. The BAC insert sequences were assembled using Roche GS *De Novo* Assembler (Newbler) V2.6 with extensive manual inspection.

### *AFGP* sequence search in Atlantic cod genome data

The Atlantic cod genome data were downloaded from http://codgenome.no/data/. The genome assemblies we used for searching AFGP cds and other analyses were: (i) the Newbler assembly ATLCOD1A, unmasked and repeat-masked; (ii) the Celera assembly ATLCOD1B; (iii) ATLCOD1C, the assembly after processing ALTCOD1A through the projection pipeline; and (iv) ALTCOD1_ANN, genome annotations including all predicted transcripts and proteins. We used our Atlantic cod Gm1-1 and polar cod *AFGP* sequences as queries to search for similar nucleotide sequence using BLASTN, and for similar translated sequences using TBLASTN. Searches utilized the default parameter settings of BLAST 2.2.24+.

## Competing interests

The authors declare no competing interests.

## Authors’ contributions

XZ performed the polar cod BAC library construction, sequencing and reconstruction of the polar cod AFGP locus, and all the sequence analyses in this study. YC constructed the partial genomic DNA library from the Øresund Atlantic cod, isolated and sequenced the AFGP gene. SEF genotyped the NEAC and NCC individuals. CHCC did the NEAC and NCC collection, genomic southern hybridization and codon usage analyses. XZ and CHCC wrote the paper. All authors read and approved the final manuscript.

## Supplementary Material

Additional file 1 **Nucleotide sequence alignment of the Gm1-1 AFGP gene [GenBank:AF529262] from the Øresund, Denmark Atlantic cod and six of the seven AFGP genes we identified from Atlantic cod genome data [**[3]**].** ATLCOD1A_AFGP7 was not included in the alignment due to long insertions in the putative intron region at 5′ of the sequence (given in lower case). ‘N’s represent gaps in the Atlantic cod sequence assembly [3]. Dashed lines indicate gaps introduced by the alignment. Asterisks indicate nucleotide identity in the column disregarding “N”. Single-letter amino acid translation of Gm1-1 is given in red, below the first nucleotide of each codon in the nucleotide alignment. ATLCOD1A_AFGP1 has the most number of amino acid substitutions (first line below Gm1-1 aa sequence), while all other ATLCOD1A AFGP sequences have few substitutions (second line below Gm1-1 amino acid sequence). Substitutions given in green would disrupt the regular (Ala/Pro-Ala-Thr) tripeptide units, and those given in blue would not. ATLCOD1A_AFGP1 has a reading frame shift at the 5′ to AFGP coding region, which would render it a pseudogene unless the frame shift reflects sequencing or assembly error. ATLCOD1A_AFGP5 and Gm1-1 are very likely counterparts in the respective individuals as their aligned sequences are 99.8% identical. The grey shaded sequences in ALTCOD1A AFGP genes were identified and masked by Star et al. using RepeatMasker with RepBase Update (teleost) TE library, and a custom library created de novo with RepeatModeler to identify novel repeats in the Atlantic cod genome (Supplementary Note 16 and Supplementary Table 6 of [3]). The repeat masking eliminated almost all partial AFGP coding sequences that remained after the initial removal of highly repetitive sequences from the Roche 454 reads prior to sequence assembly. Click here for file
