# Supplementary material for: Protein genes in repetitive sequence—antifreeze glycoproteins in Atlantic cod genome
Source: BMC Genomics. 2012 Jul 2;13:293. doi: 10.1186/1471-2164-13-293 (PMC3441883; doi:10.1186/1471-2164-13-293)
Supplement: Additional file 1 — Nucleotide sequence alignment of the Gm1-1 AFGP gene [GenBank:AF529262] from the Øresund, Denmark Atlantic cod and six of the seven AFGP genes we identified from Atlantic cod genome data [[3]]. ATLCOD1A_AFGP7 was not included in the alignment due to long insertions in the putative intron region at 5′ of the sequence (given in lower case). ‘N’s represent gaps in the Atlantic cod sequence assembly [3]. Dashed lines indicate gaps introduced by the alignment. Asterisks indicate nucleotide identity in the column disregarding “N”. Single-letter amino acid translation of Gm1-1 is given in red, below the first nucleotide of each codon in the nucleotide alignment. ATLCOD1A_AFGP1 has the most number of amino acid substitutions (first line below Gm1-1 aa sequence), while all other ATLCOD1A AFGP sequences have few substitutions (second line below Gm1-1 amino acid sequence). Substitutions given in green would disrupt the regular (Ala/Pro-Ala-Thr) tripeptide units, and those given in blue would not. ATLCOD1A_AFGP1 has a reading frame shift at the 5′ to AFGP coding region, which would render it a pseudogene unless the frame shift reflects sequencing or assembly error. ATLCOD1A_AFGP5 and Gm1-1 are very likely counterparts in the respective individuals as their aligned sequences are 99.8% identical. The grey shaded sequences in ALTCOD1A AFGP genes were identified and masked by Star et al. using RepeatMasker with RepBase Update (teleost) TE library, and a custom library created de novo with RepeatModeler to identify novel repeats in the Atlantic cod genome (Supplementary Note 16 and Supplementary Table 6 of [3]). The repeat masking eliminated almost all partial AFGP coding sequences that remained after the initial removal of highly repetitive sequences from the Roche 454 reads prior to sequence assembly. [file 1471-2164-13-293-S1.doc]

**Additional File 1.**

**Gm1-1_AFGP ggatccaaccggttcaagttgccttgatttccacttcaagaataactactacactccaaa**

ATLCOD1A_AFGP1 tgat---actc-tttacatttcagtg--ttgtagtgccaggcacacagctgctctccaaa

ATLCOD1A_AFGP6 tgatccaacct-gtcaagttgccttgatttccacttcaagaataactactacactccaaa

ATLCOD1A_AFGP3 tgaaccaaccggtt-aagttgccttgatttccacttcaagaattactactacactcca--

ATLCOD1A_AFGP4 tgatccaaccggttcaagttgccttgatttccacttcaagagtaacaactacactccaaa

ATLCOD1A_AFGP5 tgatccaaccggttcaagttgccttgatttccacttcaagaataactactacactccaaa

ATLCOD1A_AFGP2 tgatccaactggtc-aagttgccttgatttccgcttcaagaataactactacactccaaa

** ** * ** * ** ** * * ** ** ** * *****

**Gm1-1_AFGP gcaaccatggcttttctaataaggtgatttgtttattttatagttcttttctcagtgatt**

ATLCOD1A_AFGP1 gcaacaatggcttttctcttaaggtgatttgtttattttgtagttctttactctgtgatt

ATLCOD1A_AFGP6 gcaaccatggcgtttctaataaggtgatttgtttattttatagttctttactctgtgatt

ATLCOD1A_AFGP3 ---accatggcgtttctaataaggtgatttgtttattttgtagtactttactctgtgatt

ATLCOD1A_AFGP4 gcaaccatggcttttctaataaggtgatttgtttattttgtaattctttactcggtgatc

ATLCOD1A_AFGP5 gcaaccatggcttttctaataaggtgatttgtttattttatagttcttttctcagtgatt

ATLCOD1A_AFGP2 gcaaccatggcttttctaataaggtgatttgtttattttgtagttctttactcagtgatt

** ***** ***** ******************** ** * **** *** *****

**Gm1-1_AFGP aatcagtggcatggacttaatgcattacttcaaatttatctac-agggtactgcaattgg**

ATLCOD1A_AFGP1 aatcagtgaaatgcacttaatgcatttcttcaaatttctcttccagggtactgcaattag

ATLCOD1A_AFGP6 aatcaatggcatggactaaatgcatttcttcaaatttatcaaccagggtactgcaattgt

ATLCOD1A_AFGP3 aatcagtggcatggactaaatgcatttcttcaaatttatctaccagggtactgcaattgg

ATLCOD1A_AFGP4 aatcagtggcatggacttaatgcatttcttcaaatttatctaccagggtactgtaattgg

ATLCOD1A_AFGP5 aatcagtggcatggacttaatgcattacttcaaatttatctac-agggtactgcaattgg

ATLCOD1A_AFGP2 aatcagtggcatggactaaatgcatttcttcaaatttatctaccagggtactgcaattgg

***** ** *** *** ******** ********** ** * ********* ****

**Gm1-1_AFGP ctctactacttgctgtgggttctcaagtacatggtgagttga--atccaaaagtatgaaa**

ATLCOD1A_AFGP1 ctcttctacttgctgtgggttctcaagtacatggtgagttga--ttccaaaagtatgaat

ATLCOD1A_AFGP6 ctctactacttgctgtgggttctcaagtacaaggtgagttga--ctccaaaagtatgaat

ATLCOD1A_AFGP3 ctctgctacttgctgtgggttctnnnnnnnnnnnnnnnnnnn--nnnnnnnnnnnnnnnn

ATLCOD1A_AFGP4 ctctaatacttgctgttggttctnnnnnnnnnnnnnnnnnnn--nnnnnnnnnnnnnnnn

ATLCOD1A_AFGP5 ctctactacttgctgtgggttctcaagtacatggtgagttga--atccaaaagtatgaaa

ATLCOD1A_AFGP2 ctttactacttgcggtgggttctcaagtacatggtgagttgagaatccaaaagtatgaat

** * ******* ** ************** ********** **************

**Gm1-1_AFGP ttaaacaggcaggtagccatgtgctaacaagtatgcataacagcttacaggctcgtgctt**

ATLCOD1A_AFGP1 ttaaacaggcaggcagccatgtgctaacaagtatgcataacagcttacaggctcgtgctt

ATLCOD1A_AFGP6 ttaaacaggcaggtaaccatgtgctaacaagtattcataacagcttacaggctcgtgctt

ATLCOD1A_AFGP3 nnnnnnnnnnnnnnnnnnnnnnnnnnnnnnnnnnnnnnnnnnnnnnnnnnnnnnnnnnnn

ATLCOD1A_AFGP4 nnnnnnnnnnnnnnnnnnnnnnnnnnnnnnnnnnnnnnnnnnnnnnnnnnnnnnnnnnnn

ATLCOD1A_AFGP5 ttaaacaggcaggtagccatgtgctaacaagtatgcataacagcttacaggctcgtgctt

ATLCOD1A_AFGP2 ttaaacaggcaggtagccatgtgctaacaagtatgcataacagcttacaggcttgtgctt

************* * ****************** ****************** ******

**Gm1-1_AFGP gctgggccccagcagcaccagcagtctcctagctacgagcagc-accggctgctcgtagC**

ATLCOD1A_AFGP1 gctgggccccagcaacaccagcagtctccaagctacaagcagctacagaagtctcctagC

ATLCOD1A_AFGP6 gctgggccccagcagcaccagcagtctcctagctacgagcagc-accagctgctcgtagC

ATLCOD1A_AFGP3 nnnnnnnnnnnnnnnnnnnnnnnnnnnnnnnnnnnnnnnnnnnnnnnnnnnnnnnnnnnN

ATLCOD1A_AFGP4 nnnnnnnnnnnnnnnnnnnnnnnnnnnnnnnnnnnnnnnnnnnnnnnnnnnnnnnnnnnN

ATLCOD1A_AFGP5 gctgggccccagcagcaccagcagtctcctagctacgagcagc-accggctgctcgtagC

ATLCOD1A_AFGP2 gctgggcccctgcagcaccagcagtctcctagctacgagcagc-actagctgctcgtagC

********** *** ************** ****** ****** * *** ****

**Gm1-1_AFGP TAGACCAGCCGCTGCAGCCACAGCCGCCACTC--CAGCAACAGCGGCCACTCCGGCAACA**

ATLCOD1A_AFGP1 AGCAGCAGCAGCACCAGTGACAGCAGGCTCCTGGCTACAACTGcAGCCACTCCAGCACCA

ATLCOD1A_AFGP6 CAGACCAGCCGCTGCAGCCACTCCAGCC-----------ACAGCCGCCACTCCAGCAACA

ATLCOD1A_AFGP3 NNNNNNNNNNNNNNNNNNNNNNNNNNNNNNNNNNNNNNNNNNNNNNNNNNNNNNNNNNNN

ATLCOD1A_AFGP4 NNNNNNNNNNNNNNNNNNNNNNNNNNNNNNNNNNNNNNNNNNNNNNNNNNNNNNNNNNNN

ATLCOD1A_AFGP5 TAGACCAGCCGCTGCAGCCACAGCCGCCACTC--CAGCAACAGCGGCCACTCCGGCAACA

ATLCOD1A_AFGP2 CAGACAAGCCGCTGCAGCCACAGCCGCC-----------ACAGCCGCCACTCCAGCAACA

* *** ** *** ** * * * ** * ******** *** **

R P A A A A T A A T P A T A A T P A T

(location of ATLCOD1A_AFGP1 frame-shift segment) P

**Gm1-1_AFGP GCGGCCACTCCGGCAACAGCGGCCACTCCGGCCACTGCGGCCACACCGGCCGCTGCGGCC**

ATLCOD1A_AFGP1 GCAGCCACTCCAGCCTATCGAGCCAGTCCTGCCACTCCAGCCAGTCTAATAACAGCAGCC

ATLCOD1A_AFGP6 GCAGCCACTCCAGCCACAGCAGCCACTCCAGCCNNNNNNNNNNNNNNNNNNNNNNNNNNN

ATLCOD1A_AFGP3 NNNNNNNNNNNNNNNNNNNNNNNNNNNNNNNNNNNNNNNNNNNNNNNNNNNNNNNNNNNN

ATLCOD1A_AFGP4 NNNNNNNNNNNNNNNNNNNNNNNNNNNNNNNNNNNNNNNNNNNNNNNNNNNNNNNNNNNN

ATLCOD1A_AFGP5 GCGGCCACTCCGGCAACAGCGGCCACTCCGGCCACTGCGGCCACACCGGCCGCTGCGNNN

ATLCOD1A_AFGP2 GCAGCCACTCCAG------------------CCGCTGCAGCCACA---------GCGGCA

** ******** * ** ** * **** ** **

A A T P A T A A T P A T A A T P A A A A

Y R S P S L I T

A

**Gm1-1_AFGP ACAGCGGCAACTGCGGCCACAGCGGCAACTGCAGCCACAGCCGCCACAGCCGCCACCGCC**

ATLCOD1A_AFGP1 ACGACGGCCACACCAGCTACTTCAGAAACCATAGCCACTCCAGCCAATCCAGCCACTCCA

ATLCOD1A_AFGP6 NNNNNNNNNNNNNNNNNNNNNNNNNNNNNNNNNNNNNNNNNNNNNNNNNNNNNNNNNNNN

ATLCOD1A_AFGP3 NNNNNNNNNNNNNNNNNNNNNNNNNNNNNNNNNNNNNNNNNNNNNNNNNNNNNNNNNNNN

ATLCOD1A_AFGP4 NNNNNNNNNNNNNNNNNNNNNNNNNNNNNNNNNNNNNNNNNNNNNNNNNNNNNNNNNNNN

ATLCOD1A_AFGP5 NNNNNNNNNNNNNNNNNNNNNNNNNNNNNNNNNNNNNNNNNNNNNNNNNNNNNNNNNNNN

ATLCOD1A_AFGP2 ACAGCGGCCACTGCTGCCACTCTTGCCACAGCCGCCAAAGCCGCCACTGCGGCCACCGCC

** **** ** * ** ** * ** **** ** **** * ***** **

T A A T A A T A A T A A T A A T A A T A

T P S E I P N P P

K

**Gm1-1_AFGP GCCACAGCCGCCACAGCAGCCACAGCCGCCACCGCCGCCACAGCCGCCACAGCCGCCACC**

ATLCOD1A_AFGP1 GCAACAGCAGCCACTCCAGCCACTCTTGCCACTCCATCCACTCCAGCAACTCCAGTAACA

ATLCOD1A_AFGP6 NNNNNNNNNNNNNNNNNNNNNNNNNNNNNNNNNNNNNNNNNNNNNNNNNNNNNNNNNNNN

ATLCOD1A_AFGP3 NNNNNNNNNNNNNNNNNNNNNNNNNNNNNNNNNNNNNNNNNNNNNNNNNNNNNNNNNNNN

ATLCOD1A_AFGP4 NNNNNNNNNNNNNNNNNNNNNNNNNNNNNNNNNNNNNNNNNNNNNNNNNNNNNNNNNNNN

ATLCOD1A_AFGP5 NNNNNNNNNNNNNNNNNNNNNNNNNNNNNNNNNNNNNNNNNNNNNNNNNNNNNNNNNNNN

ATLCOD1A_AFGP2 GCCACAGCGGCCACAGCCGCCACCTCCACCGCC---------------------------

** ***** ***** * ***** ** *

A T A A T A A T A A T A A T A A T A A T

P L P S P P V

S T A

**Gm1-1_AFGP GCCGCCACTGCCGCCACAGCGGCAACTGCGGCAACAGCGGCCACTGCCGCCACTGCCGCC**

ATLCOD1A_AFGP1 GCAGCCACTCCAGCTACTCCAGCCACTCCAGCAACAGCAGCCACTCCAGCTACTCCAGCC

ATLCOD1A_AFGP6 NNNNNNNNNNNNNNNNNNNNNNNNNNNNNNNNNNNNNNNNNNNNNNNNNNNNNNNNNNNN

ATLCOD1A_AFGP3 NNNNNNNNNNNNNNNNNNNNNNNNNNNNNNNNNNNNNNNNNNNNNNNNNNNNNNNNNNNN

ATLCOD1A_AFGP4 NNNNNNNNNNNNNNNNNNNNNNNNNNNNNNNNNNNNNNNNNNNNNNNNNNNNNNNNNNNN

ATLCOD1A_AFGP5 NNNNNNNNNNNNNNNNNNNNNNNNNNNNNNNNNNNNNNNNNNNNNNNNNNNNNNNNNNNN

ATLCOD1A_AFGP2 ------------------------------------------------------------

A A T A A T A A T A A T A A T A A T A A

P P P P P

**Gm1-1_AFGP ACAGCAGCCACCGCAGCCACCGCCGCCACCGCCGCCACTGCCGCCACCGCCGCCACCGCT**

ATLCOD1A_AFGP1 ACTCCAGCAACTGCAGCCACTTCAGCAACTCCAGCCACTCAAGCAACATCAGCCACTCCA

ATLCOD1A_AFGP6 NNNNNNNNNNNNNNNNNNNNNNNNNNNNNNNNNNNNNNNNNNNNNNNNNNNNNNNNNNNN

ATLCOD1A_AFGP3 NNNNNNNNNNNNNNNNNNNNNNNNNNNNNNNNNNNNNNNNNNNNNNNNNNNNNNNNNNNN

ATLCOD1A_AFGP4 NNNNNNNNNNNNNNNNNNNNNNNNNNNNNNNNNNNNNNNNNNNNNNNNNNNNNNNNNNNN

ATLCOD1A_AFGP5 NNNNNNNNNNNNNNNNNNNNNNNNNNNNNNNNNNNNNNNNNNNNNNNNNNNNNNNNNNNN

ATLCOD1A_AFGP2 ------------------------------------------------------------

T A A T A A T A A T A A T A A T A A T A

P S P Q S P

**Gm1-1_AFGP GCCACAGCCGCCACTGCAGCCACTGCAGCCACAGCGGCAACTGCAGCCACAGCAGCAACT**

ATLCOD1A_AFGP1 GCCAAAGCAGCCACTGCAGCCACTACAAAAACTCCAGCAACAGCAGCAACCCCAGCAACA

ATLCOD1A_AFGP6 NNNNNNNNNNNNNNNNNNNNNNNNNNNNNNNNNNNNNNNNNNNNNNNNNNNNNNNNNNNN

ATLCOD1A_AFGP3 NNNNNNNNNNNNNNNNNNNNNNNNNNNNNNNNNNNNNNNNNNNNNNNNNNNNNNNNNNNN

ATLCOD1A_AFGP4 NNNNNNNNNNNNNNNNNNNNNNNNNNNNNNNNNNNNNNNNNNNNNNNNNNNNNNNNNNNN

ATLCOD1A_AFGP5 NNNNNNNNNNNNNNNNNNNNNNNNNNNNNNNNNNNNNNNNNNNNNNNNNNNNNNNNNNNN

ATLCOD1A_AFGP2 ------------------------------------------------------------

A T A A T A A T A A T A A T A A T A A T

K T K P P

**Gm1-1_AFGP CCAGCAAGAGCAGCCACTCCGGCCACAGCGGCCACAGCGGCAACTGCCGCCACTGCCGCC**

ATLCOD1A_AFGP1 GCAGCAACTCCAGCCACTCCAGCAACAGCAGCCACTCCAGCCACTCGTGCCACTCCTGCC

ATLCOD1A_AFGP6 NNNNNNNNNNNNNNNNNNNNNNNNNNNNNNNNNNNNNNNNNNNNNNNNNNNNNNNNNNNN

ATLCOD1A_AFGP3 NNNNNNNNNNNNNNNNNNNNNNNNNNNNNNNNNNNNNNNNNNNNNNNNNNNNNNNNNNNN

ATLCOD1A_AFGP4 NNNNNNNNNNNNNNNNNNNNNNNNNNNNNNNNNNNNNNNNNNNNNNNNNNNNNNNNNNNN

ATLCOD1A_AFGP5 NNNNNNNNNNNNNNNNNNNNNNNNNNNNNNNNNNNNNNNNNNNNNNNNNNNNNNNNNNNN

ATLCOD1A_AFGP2 ------------------------------------------------------------

P A R A A T P A T A A T A A T A A T A A

A T P P R P

**Gm1-1_AFGP ACAGCAGCCACCGCCGCCACAGCCGCCACCGCCGCCACAGCCGCCACAGCCGCCACCGCC**

ATLCOD1A_AFGP1 ACTCCAGCTACTCCAGCCACTCCTGCCACTCCAGCAACACCAGCCACTCCAGCCACTCCT

ATLCOD1A_AFGP6 NNNNNNNNNNNNNNNNNNNNNNNNNNNNNNNNNNNNNNNNNNNNNNNNNNNNNNNNNNNN

ATLCOD1A_AFGP3 NNNNNNNNNNNNNNNNNNNNNNNNNNNNNNNNNNNNNNNNNNNNNNNNNNNNNNNNNNNN

ATLCOD1A_AFGP4 NNNNNNNNNNNNNNNNNNNNNNNNNNNNNNNNNNNNNNNNNNNNNNNNNNNNNNNNNNNN

ATLCOD1A_AFGP5 NNNNNNNNNNNNNNNNNNNNNNNNNNNNNNNNNNNNNNNNNNNNNNNNNNNNNNNNNNNN

ATLCOD1A_AFGP2 ------------------------------------------------------------

T A A T A A T A A T A A T A A T A A T A

P P P P P P P

**Gm1-1_AFGP GCCACTGCAGCCACAGCTGCAACTGCAGCCACAGCAGCAACTCCAGCAAGAGCAGCCACT**

ATLCOD1A_AFGP1 GCCACTCCAGCAACACCTGCCACTCCAGCCACTCCTGCCACTCCAGCCACACCAGCCACT

ATLCOD1A_AFGP6 NNNNNNNNNNNNNNNNNNNNNNNNNNNNNNNNNNNNNNNNNNNNNNNNNNNNNNNNNNNN

ATLCOD1A_AFGP3 NNNNNNNNNNNNNNNNNNNNNNNNNNNNNNNNNNNNNNNNNNNNNNNNNNNNNNNNNNNN

ATLCOD1A_AFGP4 NNNNNNNNNNNNNNNNNNNNNNNNNNNNNNNNNNNNNNNNNNNNNNNNNNNNNNNNNNNN

ATLCOD1A_AFGP5 NNNNNNNNNNNNNNNNNNNNNNNNNNNNNNNNNNNNNNNNNNNNNNNNNNNNNNNNNNNN

ATLCOD1A_AFGP2 ------------------------------------------------------------

A T A A T A A T A A T A A T P A R A A T

P P P P T P

**Gm1-1_AFGP CCGGCAACAGCGGCCACTCCGGCCACAGCGGCCGCTGCCGCCACAGCCGCCACAGCGGCA**

ATLCOD1A_AFGP1 CCTGCCACTGCNNNNNNNNNNNNNNNNNNNNNNNNNNNNNNNNNNNNNNNNNNNNNNNNN

ATLCOD1A_AFGP6 NNNNNNNNNNNNNNNNNNNNNNNNNNNNNNNNNNNNNNNNNNNNNNNNNNNNNNNNNNNN

ATLCOD1A_AFGP3 NNNNNNNNNNNNNNNNNNNNNNNNNNNNNNNNNNNNNNNNNNNNNNNNNNNNNNNNNNNN

ATLCOD1A_AFGP4 NNNNNNNNNNNNNNNNNNNNNNNNNNNNNNNNNNNNNNNNNNNNNNNNNNNNNNNNNNNN

ATLCOD1A_AFGP5 NNNNNNNNNNNNNNNNNNNNNNNNNNNNNNNNNNNNNNNNNNNNNNNNNNNNNNNNNNNN

ATLCOD1A_AFGP2 ------------------------------------------------------------

P A T A A T P A T A A A A A T A A T A A

**Gm1-1_AFGP ACTGCGGCAACAGCCGCCACTGCCGCCACCGCCGCCACCGCAGCCACCGCAGCCACCGCA**

ATLCOD1A_AFGP1 NNNNNNNNNNNNNNNNNNNNNNNNNNNNNNNNNNNNNNNNNNNNNNNNNNNNNNNNNNNN

ATLCOD1A_AFGP6 NNNNNNNNNNNNNNNNNNNNNNNNNNNNNNNNNNNNNNNNNNNNNNNNNNNNNNNNNNNN

ATLCOD1A_AFGP3 NNNNNNNNNNNNNNNNNNNNNNNNNNNNNNNNNNNNNNNNNNNNNNNNNNNNNNNNNNNN

ATLCOD1A_AFGP4 NNNNNNNNNNNNNNNNNNNNNNNNNNNNNNNNNNNNNNNNNNNNNNNNNNNNNNNNNNNN

ATLCOD1A_AFGP5 NNNNNNNNNNNNNNNNNNNNNNNNNNNNNNNNNNNNNNNNNNNNNNNNNNNNNNNNNNNN

ATLCOD1A_AFGP2 ------------------------------------------------------------

T A A T A A T A A T A A T A A T A A T A

**Gm1-1_AFGP GCCACTGCCGCCACAGCCGCCACAGCGGCAACAGCGGCAACTGCCGCCACTGCCGCCACA**

ATLCOD1A_AFGP1 NNNNNNNNNNNNNNNNNNNNNNNNNNNNNNNNNNNNNNNNNNNNNNNNNNNNNNNNNNNN

ATLCOD1A_AFGP6 NNNNNNNNNNNNNNNNNNNNNNNNNNNNNNNNNNNNNNNNNNNNNNNNNNNNNNNNNNNN

ATLCOD1A_AFGP3 NNNNNNNNNNNNNNNNNNNNNNNNNNNNNNNNNNNNNNNNNNNNNNNNNNNNNNNNNNNN

ATLCOD1A_AFGP4 NNNNNNNNNNNNNNNNNNNNNNNNNNNNNNNNNNNNNNNNNNNNNNNNNNNNNNNNNNNN

ATLCOD1A_AFGP5 NNNNNNNNNNNNNNNNNNNNNNNNNNNNNNNNNNNNNNNNNNNNNNNNNNNNNNNNNNNN

ATLCOD1A_AFGP2 ------------------------------------------------------------

A T A A T A A T A A T A A T A A T A A T

**Gm1-1_AFGP GCAGCCACCGCAGCCACCGCCGCCACAGCCGCCACAGCCGCCACCGCAGCCACAGCCGCC**

ATLCOD1A_AFGP1 NNNNNNNNNNNNNNNNNNNNNNNNNNNNNNNNNNNNNNNNNNNNNNNNNNNNNNNNNNNN

ATLCOD1A_AFGP6 NNNNNNNNNNNNNNNNNNNNNNNNNNNNNNNNNNNNNNNNNNNNNNNNNNNNNNNNNNNN

ATLCOD1A_AFGP3 NNNNNNNNNNNNNNNNNNNNNNNNNNNNNNNNNNNNNNNNNNNNNNNNNNNNNNNNNNNN

ATLCOD1A_AFGP4 NNNNNNNNNNNNNNNNNNNNNNNNNNNNNNNNNNNNNNNNNNNNNNNNNNNNNNNNNNNN

ATLCOD1A_AFGP5 NNNNNNNNNNNNNNNNNNNNNNNNNNNNNNNNNNNNNNNNNNNNNNNNNNNNNNNNNNNN

ATLCOD1A_AFGP2 ------------------------------------------------------------

A A T A A T A A T A A T A A T A A T A A

**Gm1-1_AFGP ACTGCCGCCACTGCAGCCACTGCAGCCACAGCAGCAACTGCAGCCACAGCAGCAACTCCA**

ATLCOD1A_AFGP1 NNNNNNNNNNNNNNNNNNNNNNNNNNNNNNNNNNNNNNNNNNNNNNNNNNNNNNNNNNNN

ATLCOD1A_AFGP6 NNNNNNNNNNNNNNNNNNNNNNNNNNNNNNNNNNNNNNNNNNNNNNNNNNNNNNNNNNNN

ATLCOD1A_AFGP3 NNNNNNNNNNNNNNNNNNNNNNNNNNNNNNNNNNNNNNNNNNNNNNNNNNNNNNNNNNNN

ATLCOD1A_AFGP4 NNNNNNNNNNNNNNNNNNNNNNNNNNNNNNNNNNNNNNNNNNNNNNNNNNNNNNNNNNNN

ATLCOD1A_AFGP5 NNNNNNNNNNNNNNNNNNNNNNNNNNNNNNNNNNNNNNNNNNNNNNNNNNNNNNNNNNNN

ATLCOD1A_AFGP2 ------------------------------------------------------------

T A A T A A T A A T A A T A A T A A T P

**Gm1-1_AFGP GCAAGAGCAGCCACTCCGGCAACAGCGGCCACTCCGGCCACAGCGGCCGCTGCCGCCACT**

ATLCOD1A_AFGP1 NNNNNNNNNNNNNNNNNNNNNNNNNNNNNNNNNNNNNNNNNNNNNNNNNNNNNNNNNNNN

ATLCOD1A_AFGP6 NNNNNNNNNNNNNNNNNNNNNNNNNNNNNNNNNNNNNNNNNNNNNNNNNNNNNNNNNNNN

ATLCOD1A_AFGP3 NNNNNNNNNNNNNNNNNNNNNNNNNNNNNNNNNNNNNNNNNNNNNNNNNNNNNNNNNNNN

ATLCOD1A_AFGP4 NNNNNNNNNNNNNNNNNNNNNNNNNNNNNNNNNNNNNNNNNNNNNNNNNNNNNNNNNNNN

ATLCOD1A_AFGP5 NNNNNNNNNNNNNNNNNNNNNNNNNNNNNNNNNNNNNNNNNNNNNNNNNNNNNNNNNNNN

ATLCOD1A_AFGP2 ------------------------------------------------------------

A R A A T P A T A A T P A T A A A A A T

**Gm1-1_AFGP GCCGCCACCGCCGCCACAGCAGCCACCGCGGCCACAGCCGCCACAGCCGCCACAGCGGCA**

ATLCOD1A_AFGP1 NNNNNNNNNNNNNNNNNNNNNNNNNNNNNNNNNNNNNNNNNNNNNNNNNNNNNNNNNNNN

ATLCOD1A_AFGP6 NNNNNNNNNNNNNNNNNNNNNNNNNNNNNNNNNNNNNNNNNNNNNNNNNNNNNNNNNNNN

ATLCOD1A_AFGP3 NNNNNNNNNNNNNNNNNNNNNNNNNNNNNNNNNNNNNNNNNNNNNNNNNNNNNNNNNNNN

ATLCOD1A_AFGP4 NNNNNNNNNNNNNNNNNNNNNNNNNNNNNNNNNNNNNNNNNNNNNNNNNNNNNNNNNNNN

ATLCOD1A_AFGP5 NNNNNNNNNNNNNNNNNNNNNNNNNNNNNNNNNNNNNNNNNNNNNNNNNNNNNNNNNNNN

ATLCOD1A_AFGP2 ------------------------------------------------------------

A A T A A T A A T A A T A A T A A T A A

**Gm1-1_AFGP ACAGCGGCCACAGCGGCCACCGCGGCCACAGCCGCCACAGCGGCCACAGCCGCCACAGCG**

ATLCOD1A_AFGP1 NNNNNNNNNNNNNNNNNNNNNNNNNNNNNNNNNNNNNNNNNNNNNNNNNNNNNNNNNNNN

ATLCOD1A_AFGP6 NNNNNNNNNNNNNNNNNNNNNNNNNNNNNNNNNNNNNNNNNNNNNNNNNNNNNNNNNNNN

ATLCOD1A_AFGP3 NNNNNNNNNNNNNNNNNNNNNNNNNNNNNNNNNNNNNNNNNNNNNNNNNNNNNNNNNNNN

ATLCOD1A_AFGP4 NNNNNNNNNNNNNNNNNNNNNNNNNNNNNNNNNNNNNNNNNNNNNNNNNNNNNNNNNNNN

ATLCOD1A_AFGP5 NNNNNNNNNNNNNNNNNNNNNNNNNNNNNNNNNNNNNNNNNNNNNNNNNNNNNNNNNNNN

ATLCOD1A_AFGP2 ------------------------------------------------------------

T A A T A A T A A T A A T A A T A A T A

**Gm1-1_AFGP GCAACAGCGGCCACAGCCGCCACTGCGGCCACAGCGGCCACCGCAGCCACCGCAGCCACT**

ATLCOD1A_AFGP1 NNNNNNNNNNNNNNNNNNNNNNNNNNNNNNNNNNNNNNNNNNNNNNNNNNNNNNNNNNNN

ATLCOD1A_AFGP6 NNNNNNNNNNNNNNNNNNNNNNNNNNNNNNNNNNNNNNNNNNNNNNNNNNNNNNNNNNNN

ATLCOD1A_AFGP3 NNNNNNNNNNNNNNNNNNNNNNNNNNNNNNNNNNNNNNNNNNNNNNNNNNNNNNNNNNNN

ATLCOD1A_AFGP4 NNNNNNNNNNNNNNNNNNNNNNNNNNNNCCACCGCCGCCACAGCAGCCACCGCGGCCACC

ATLCOD1A_AFGP5 NNNNNNNNNNNNNNNNNNNNNNNNNNNNNNNNNNNNNNNNNNNNNNNNNNNNNNNNNNNN

ATLCOD1A_AFGP2 ------------------------------------------------------------

**** ** ***** *********** *****

A T A A T A A T A A T A A T A A T A A T

**Gm1-1_AFGP GCAGCCACCGCAGCCACTGCAGCCACTGCCGCCACAGCGGCAACAGCGGCAACTCCAGCA**

ATLCOD1A_AFGP1 NNNNNNNNNNNNNNNNNNNNNNNNNNNNNNNNNNNNNNNNNNNNNNNNNNNNNNNNNNNN

ATLCOD1A_AFGP6 NNNNNNNNNNNNNNNNNNNNNNNNNNNNNNNNNNNNNNNNNNNNNNNNNNNNNNNNNNNN

ATLCOD1A_AFGP3 NNNNNNNNNNNNNNNNNNNNNNNNNNNNNNNNNNNNNNNNNNNNNNNNNNNNNNNNNNNN

ATLCOD1A_AFGP4 GCAGCCACCTCGGCCACAGCAGCCACTGCAGCCACAGCCGCCACTGAAGCCACAGCAGCC

ATLCOD1A_AFGP5 NNNNNNNNNNNNNNNNNNNNNNNNNNNNNNNNNNNNNNNNNNNNNNNNNNNNNNCCAGCA

ATLCOD1A_AFGP2 ---------------------------------------------------------GCC

********* * ***** *********** ******** ** ** * ** ** ****

A A T A A T A A T A A T A A T A A T P A

**Gm1-1_AFGP AGAGCAGCCACTCCGGCAACAGCAGCCACTCCGGCCACAGCAGCCACCCCAGCCACAGCA**

ATLCOD1A_AFGP1 NNNNNNNNNNNNNNNNNNNNNNNNNNNNNNNNNNNNNNNNNNNNNNNNNNNNNNNNNNNN

ATLCOD1A_AFGP6 NNNNNNNNNNNNNNNNNNNNNNNNNNNNNNNNNNNNNNNNNNNNNNNNNNNNNNNNNNNN

ATLCOD1A_AFGP3 NNNNNAGCAACTCCAGCAAGAGCAGCCACTCCGGCAACAGCAGCCACTCCAGCCACAGCA

ATLCOD1A_AFGP4 ACAGCACCAACTCCAGCAAGAGCAGCCACTCCGGCAACAGCAGCCACTCCGGCCACAGCA

ATLCOD1A_AFGP5 AGAGCAGCCACTCCGGCAACAGCAGCCACTCCGGCCACAGCAGCCACCCCAGCCACAGCA

ATLCOD1A_AFGP2 ACAGCAGCAACTCCAGCAAGAGCAGCCACTCCGGCCAGAGCAGCCACCCCAGCCACAGCA

* ****** ***** **** *************** * ********* ** *********

R A A T P A T A A T P A T A A T P A T A

**Gm1-1_AFGP GCCACACCAGCAGCAGCAGTCCTCTAGctaccagccccattataaatggcagcagcagtc**

ATLCOD1A_AFGP1 NNNNNNNNNNNNNNNNNNNNNNNNNNNnnnnnnnnnnnnnnnnnnnnnnnnnnnnnnnnn

ATLCOD1A_AFGP6 NNNNNNNNNNNNNNNNNNNNNNNNNNNnnnnnnnnnnnnnnnnnnnnnnnnnnnnnnnnn

ATLCOD1A_AFGP3 GCCACTCCTGCAGCAGCAGTCCTCTAGctaccagccccagtagaaatg----tagcagtc

ATLCOD1A_AFGP4 GCAACTCCAGCAGCAGCAGTCCTCTAGctaccagccccagtagaattggcagcagcagtc

ATLCOD1A_AFGP5 GCCACACCAGCAGCAGCAGTCCTCTAGctaccagccccattataaatggcagcagcagtc

ATLCOD1A_AFGP2 GCCAATCCTGCAGCAGCAGTCCTCTAGctaccagccccagtagaattggcagcagcagtc

** * ** ****************************** ** ** ** *******

A T P A A A V L *

**Gm1-1_AFGP tccttgatacaagcaggttgacttgttaactactctccttgtttcaaatgtcccaggcaa**

ATLCOD1A_AFGP1 nnnnnnnnnnnnnnnnnnnnnnnnnnnnnnnnnnnnnnnnnnnnnnnnnnnnnnnnnnnn

ATLCOD1A_AFGP6 nnnnnnnnnnnnnnnnnnnnnnnnnnnnnnnnnnnnnnnnnnnnnnnnnnnnnnnnnnnn

ATLCOD1A_AFGP3 tccttgctacaagcagattgacatattatctactctccttatttcaaatgtctcaggcac

ATLCOD1A_AFGP4 tccttgctacaagcaggttgacttattatctgctcttcttatttcaaatgtctgaggcac

ATLCOD1A_AFGP5 tccttgatacaagcaggttgacttgttaactactctccttgtttcaaatgtcccaggcaa

ATLCOD1A_AFGP2 tccttgctacaagcaggttgacttattatctactctccttatttcaaatgtctcaggcac

****** ************ ** * *** ** **** *** *********** *****

**Gm1-1_AFGP aataataaaatcataaatttcaaactgtctgtttattgtatgtaagctagtgtgaatgcc**

ATLCOD1A_AFGP1 nnnnnnnnnnnnnnnnnnnnnnnnnnnnnnnnnnnnnnnnnnnnnnnnnnnnnnnnnnnn

ATLCOD1A_AFGP6 nnnnnnnnnnnnnnnnnnnnnnnnnnnnnnnnnnnnnnnnnnnnnnnnnnnnnnnnnnnn

ATLCOD1A_AFGP3 aataataaaatcatcaatttcaaactgtctgtgtattgtatataagctattctgaatgcc

ATLCOD1A_AFGP4 aataataaaatcatcaatttcaaactgtctgtgtcttgtatataagctattctgaatgcc

ATLCOD1A_AFGP5 aataataaaatcataaatttcaaactgtctgtttattgtatgtaagctagtctgaatgcc

ATLCOD1A_AFGP2 aataataaaatcatcaatttcaaactgtctgtgtattgtatattagctagtccgaatgcc

************** ***************** * ****** * ***** * *******

**Gm1-1_AFGP agca**

ATLCOD1A_AFGP1 nnnn

ATLCOD1A_AFGP6 nnnn

ATLCOD1A_AFGP3 agca

ATLCOD1A_AFGP4 agca

ATLCOD1A_AFGP5 agca

ATLCOD1A_AFGP2 agca

****

**Additional File 1 legend.** Nucleotide sequence alignment of the Gm1-1 AFGP gene (bolded) [GenBank: AF529262] from the Øresund, Denmark Atlantic cod and six of the seven AFGP genes we identified from Atlantic cod genome data [3]. ATLCOD1A_AFGP7 was not included in the alignment due to long insertions in the putative intron region at 5’ of the sequence (given in lower case). ‘N’s represent gaps in the Atlantic cod sequence assembly [3]. Dashed lines indicate gaps introduced by the alignment. Asterisks indicate nucleotide identity in the column disregarding “N”. Single-letter amino acid translation of Gm1-1 is given in red, below the first nucleotide of each codon in the nucleotide alignment. ATLCOD1A_AFGP1 has the most number of amino acid substitutions (first line below Gm1-1 aa sequence), while all other ATLCOD1A AFGP sequences have few substitutions (second line below Gm1-1 amino acid sequence). Substitutions given in green would disrupt the regular (Ala/Pro-Ala-Thr) tripeptide units, and those given in blue would not. ATLCOD1A_AFGP1 has a reading frame shift at the 5’ to AFGP coding region, which would render it a pseudogene unless the frame shift reflects sequencing or assembly error. ATLCOD1A_AFGP5 and Gm1-1 are very likely counterparts in the respective individuals as their aligned sequences are 99.8% identical. The grey shaded sequences in ALTCOD1A AFGP genes were identified and masked by Star *et al* using RepeatMasker with RepBase Update (teleost) TE library, and a custom library created *de novo* with RepeatModeler to identify novel repeats in the Atlantic cod genome [3, Supplementary Note 16 and Supplementary Table 6]. The repeat masking eliminated almost all partial AFGP coding sequences that remained after the initial removal of highly repetitive sequences from the Roche 454 reads prior to sequence assembly.
